# Non-invasive vagus nerve magnetic stimulation combined with rehabilitation training for vocal cord paralysis and dysphagia following brain injury: a prospective case series study

**DOI:** 10.3389/fneur.2026.1807577

**Published:** 2026-07-01

**Authors:** Shujuan Huang, Hanbo Chen, Caixia Ouyang, Huimin Han, Yong Luo, Weifeng Wen, Lirong Liu, Xiao Lu

**Affiliations:** 1 Department of Rehabilitation Therapy, Guangdong Sanjiu Brain Hospital, Guangzhou, China; 2 Department of Rehabilitation Medicine, Guangdong Sanjiu Brain Hospital, Guangzhou, China

**Keywords:** case series, dysphagia, non-invasive magnetic stimulation, vagus nerve, vocal cord paralysis

## Abstract

**Background:**

Vocal cord paralysis combined with dysphagia following brain injury is a severe complication associated with aspiration pneumonia, asphyxiation, and malnutrition, for which traditional rehabilitation yields limited efficacy. Non-invasive vagus nerve magnetic stimulation (nVNMS) is an emerging neuromodulation technology; however, systematic clinical studies addressing this specific dual-deficit population remain lacking.

**Methods:**

A prospective case series study was conducted at Guangdong Sanjiu Brain Hospital (June 2024–May 2025), consecutively enrolling 38 inpatients with brain injury–related vocal cord paralysis and dysphagia. All patients received a 21-day combined protocol comprising left mastoid nVNMS (5 Hz, 20 min/session, once daily), low-frequency pulse electrical stimulation for swallowing (20 min/session, once daily), and manual swallowing training (30 min/session, once daily). Outcomes were assessed at baseline and post-treatment using the Standardized Swallowing Assessment (SSA), Food Intake Level Scale (FILS), Penetration-Aspiration Scale (PAS), and laryngoscopy, with adverse events recorded throughout.

**Results:**

After treatment, SSA scores decreased from 36.92 ± 4.71 to 32.63 ± 7.10 (*p* < 0.001, Cohen’s d = 0.72, large effect), FILS scores increased from 2 (2–2) to 3 (2–3) (p < 0.001, representing transition from total tube-feeding dependence to eligibility for supervised minimal oral intake), and PAS scores decreased from 6 (4–6) to 4 (4–4) (*p* < 0.001, *r* = 0.64, large effect, representing restoration of functional airway protective response). The incidence of vocal cord paralysis decreased from 100 to 63.2% (*p* < 0.001), tracheostomy cannula retention rate decreased from 55.3 to 26.3% (*p* = 0.001), with 11 patients achieving decannulation and thereby regaining airway independence; nasogastric tube retention rate decreased from 100 to 68.4% (*p* < 0.001), with 12 patients recovering oral feeding ability. No serious adverse events occurred during treatment.

**Conclusion:**

As a preliminary case series without a control group, nVNMS combined with rehabilitation training may improve swallowing safety and vocal cord function and reduce airway management requirements in patients following brain injury, with favorable short-term safety. Owing to the lack of a control group, the specific effect of nVNMS and the impact of natural recovery cannot be differentiated; thus, multicenter randomized controlled trials are required for further validation.

## 1 Introduction

Vocal cord paralysis combined with dysphagia is a common complication following brain injury. The reported incidence of dysphagia after brain injury ranges from 27% to 82% (1, 2), with concurrent vocal cord paralysis occurring in 7%–12% of cases (3). This condition can lead to aspiration pneumonia (4) (with an incidence as high as 40%), asphyxiation, and malnutrition, seriously affecting patient prognosis and quality of life. In patients with brain injury, infratentorial lesions (such as lateral medullary syndrome and brainstem hemorrhage) can directly damage the vagus nerve nuclei or fiber tracts, resulting in ipsilateral vocal cord paralysis and swallowing muscle coordination disorders. Traditional rehabilitation training has limited efficacy in patients with severe functional impairment, with effectiveness rates of only 25%–45% (5, 6). Conventional swallowing electrical stimulation combined with exercise therapy has been reported to improve swallowing safety and laryngeal function in patients with brain injury (7–9). While invasive surgical procedures (such as vocal cord injection laryngoplasty and arytenoidectomy) can improve glottic closure or opening function, they carry risks of surgical trauma and complications (10, 11). Although long-term tracheostomy and tube feeding can sustain life, they cannot fundamentally restore neural function and severely impact patient quality of life (12, 13). Therefore, exploring safe and effective non-invasive neuromodulation treatment methods has important clinical significance.

In recent years, neuromodulation technologies have shown potential in treating dysphagia following brain injury. Transcranial magnetic stimulation (TMS) and transcutaneous nerve electrical stimulation have been proven to improve swallowing function after stroke (14–16). Vagus nerve stimulation (VNS), as an established neuromodulation approach, was initially applied in the treatment of refractory epilepsy and depression. However, traditional VNS predominantly involves invasive implantable devices, requiring surgical implantation of electrodes into the cervical vagus nerve trunk, which is expensive and carries surgical risks, limiting its widespread application in the rehabilitation field (17, 18). With the rapid development of non-invasive vagus nerve stimulation technologies, transcutaneous auricular VNS (taVNS), which stimulates the auricular branch of the vagus nerve through the auricle, has demonstrated efficacy in multiple neurological disorders (19). Research by Wang Y et al. showed that taVNS can shorten swallowing reflex latency and improve swallowing safety in stroke patients with dysphagia (14). Studies have found that non-invasive vagus nerve stimulation combined with rehabilitation training can improve patients’ swallowing function scores (19).

Non-invasive vagus nerve magnetic stimulation (nVNMS) is a novel neuromodulation technology that activates afferent fibers of the pharyngeal branch of the vagus nerve by placing a magnetic stimulation coil on the body surface of the left mastoid region, utilizing pulsed magnetic fields to penetrate skin and tissue (20, 21). The pharyngeal branch of the vagus nerve courses subcutaneously in the mastoid region, carrying sensory fibers that project to the nucleus tractus solitarius, which is an important component of the medullary swallowing center. Meanwhile, the recurrent laryngeal nerve, arising from the main vagus nerve trunk, innervates vocal cord movement (6, 21–23). Theoretically, nVNMS can modulate the medullary swallowing-respiration coordination center by activating vagal afferent pathways, promote recovery of recurrent laryngeal nerve function, and enhance swallowing reflexes through the vagus nerve-nucleus tractus solitarius-nucleus ambiguus pathway.

Compared with invasive VNS, nVNMS offers advantages including non-invasiveness, safety, repeatability, and high patient compliance. Compared with auricular electrical stimulation, magnetic stimulation has greater penetration depth (up to 2–3 cm), potentially activating deep nerve fibers more effectively (24–26). However, existing VNS research has mainly focused on invasive implantable devices or auricular electrical stimulation, with a lack of systematic clinical studies on non-invasive magnetic stimulation applications for the specific population of brain injury patients with vocal cord paralysis combined with dysphagia. Particularly for patients with concurrent vocal cord movement disorders and swallowing dysfunction, whether nVNMS can simultaneously improve both functions remains unclear (14, 19, 20). This prospective case series study aims to provide, to our knowledge, one of the earliest systematic clinical evaluations of the clinical outcomes and safety of a combined intervention protocol incorporating left mastoid nVNMS alongside standard swallowing rehabilitation in patients with vocal cord paralysis and dysphagia following brain injury, exploring its multidimensional effects on swallowing function, vocal cord mobility, and removal of tracheostomy cannulas and nasogastric tubes. This will provide preliminary evidence-based support for the clinical application of this treatment modality and lay the foundation for the design of subsequent randomized controlled trials.

## 2 Materials and methods

This study was approved by the Ethics Committee of Guangdong Sanjiu Brain Hospital (Approval No: 2024-01-034). This was a single-center prospective pilot and feasibility case series study conducted in the Department of Rehabilitation Medicine at Guangdong Sanjiu Brain Hospital from June 12, 2024, to May 11, 2025. The sample size (*n* = 38) was determined by practical recruitment feasibility. All consecutive inpatients meeting the eligibility criteria during the 12-month study period (June 2024 to May 2025) were enrolled. This represents the maximum achievable sample within the constraints of a single specialized center. No eligible patients were excluded for non-clinical reasons. As this study explored a novel intervention in a population for which no prior randomized controlled data or established effect size estimates were available, a formal *a priori* power calculation was not performed. For pilot and feasibility studies conducted without prior effect size data, methodological consensus suggests that samples of 12–30 participants are typically sufficient for preliminary efficacy estimation (27, 28); the present sample of *n* = 38 exceeds this threshold. The large effect sizes observed in the study (Cohen’s d = 0.72 for SSA improvement; *r* = 0.64 for PAS improvement) provide empirical estimates to inform formal power calculations for future randomized controlled trials. This study aimed to evaluate the clinical efficacy of non-invasive vagus nerve magnetic stimulation in patients with vocal cord paralysis and dysphagia following brain injury. All patients (or their legal guardians) signed written informed consent before enrollment.

As this was a consecutive enrollment study conducted within a specialized inpatient rehabilitation unit, all inpatients admitted to the Department of Rehabilitation Medicine during the study period (June 12, 2024 to May 11, 2025) were screened for eligibility according to the pre-specified inclusion and exclusion criteria; no eligible patients were excluded for non-clinical reasons, and the 38 enrolled patients represent the complete eligible population identified during the study window. The enrolled cohort comprised 6 cases (15.8%) with unilateral vocal cord paralysis and 32 cases (84.2%) with bilateral vocal cord paralysis. All 38 enrolled patients completed the full 21-day treatment protocol and the day-28 follow-up assessment, with no loss to follow-up or dropout cases. Inclusion criteria included: (1) brain injury diagnosed by cranial CT or MRI imaging examination (including cerebral hemorrhage, cerebral infarction, traumatic brain injury, or other brain lesions), with complete imaging data and clinical medical records; (2) brain injury confirmed as the etiology of vocal cord paralysis and dysphagia through clinical assessment and neuroanatomical analysis; (3) unilateral or bilateral vocal cord paralysis confirmed by laryngoscopy; (4) dysphagia confirmed by fiberoptic endoscopic evaluation of swallowing; (5) age 18–65 years; (6) able to tolerate non-invasive magnetic stimulation treatment; (7) no prior surgical treatment for vocal cord paralysis; (8) signed informed consent. Exclusion criteria included: left mastoid skin damage, infection, or bone defects affecting coil placement; concurrent intracranial malignant tumors; intracranial metallic implants, non-diamagnetic drainage tubes, or cardiac pacemakers; previous transcranial or peripheral magnetic stimulation treatment; history of epilepsy or idiopathic epilepsy in first-degree relatives; presence of ear diseases in the left ear.

All patients completed cranial CT or MRI examination before enrollment to confirm the diagnosis and lesion location of brain injury. The diagnosis of cerebral hemorrhage and cerebral infarction followed the standards of the “Chinese Guidelines for the Diagnosis and Treatment of Acute Ischemic Stroke” and “Chinese Guidelines for the Diagnosis and Treatment of Cerebral Hemorrhage.” The diagnosis of traumatic brain injury was based on clear history of head trauma and corresponding abnormal imaging findings. Lesion locations were interpreted and classified by neuroradiologists as supratentorial, infratentorial, or mixed lesions. Lesion location determination: All patients had brain injury lesion locations determined through cranial CT or MRI imaging examination. Lesion location classification criteria were as follows: (1) Supratentorial lesions: lesions located entirely above the tentorium cerebelli, including cerebral hemispheres, basal ganglia, thalamus, and supratentorial white matter; (2) Infratentorial lesions: lesions located entirely below the tentorium cerebelli, including the brainstem (midbrain, pons, medulla oblongata) and cerebellum; (3) Mixed lesions: lesions involving both supratentorial and infratentorial regions. Lesion locations were determined by a multidisciplinary team consisting of neuroradiologists, neurologists, and rehabilitation physicians through systematic neuroimaging assessment. For each patient, after identifying all lesion sites, classification was performed according to the “culprit lesion” principle: that is, anatomical locations most likely to cause vocal cord paralysis and dysphagia were classified based on neuroanatomical pathway analysis and clinical relevance.

Special considerations for traumatic brain injury patients (6 cases, 15.8%): The injury pattern in traumatic brain injury patients may include diffuse axonal injury, multiple contusions and lacerations, or composite injuries spanning supratentorial and infratentorial regions. The following methods were used for lesion location determination: (1) All injury sites were identified through comprehensive neuroimaging examination (CT and/or MRI), including cortical contusions and lacerations, subcortical lesions, brainstem injuries, diffuse axonal injury, etc.; (2) Classification was based on the primary injury sites affecting lower cranial nerve pathways including the vagus nerve and glossopharyngeal nerve, and swallowing-related neural networks, with emphasis on evaluating brainstem (especially medulla oblongata and pons) involvement and its neuroanatomical association with vocal cord paralysis and dysphagia; (3) Patients with purely supratentorial or infratentorial injuries were classified into corresponding categories; (4) Patients with diffuse axonal injury or multiple contusions and lacerations involving both supratentorial and infratentorial structures were classified as mixed lesions; (5) Classification decisions were determined through multidisciplinary team consensus review. The distribution of lesion locations among the 6 traumatic brain injury patients in this study was: 2 cases with supratentorial lesions (bilateral frontal lobe contusions with insular-frontal opercular involvement), 1 case with infratentorial lesion (isolated pontomedullary junction contusion), and 3 cases with mixed lesions (diffuse axonal injury involving supratentorial and infratentorial structures, or multiple contusions spanning both regions). All patients completed systematic clinical assessment upon enrollment. Laryngoscopy was performed by a swallowing specialist physician with over 20 years of experience. Swallowing function assessment was completed through fiberoptic endoscopic evaluation of swallowing (FEES) examination. FEES examination was conducted according to standardized procedures, including: (1) laryngopharyngeal anatomical structure assessment; (2) cough reflex assessment (mechanical stimulation of the posterior pharyngeal wall); (3) secretion management assessment; (4) swallowing trials with different food consistencies (liquid, puree, solid); (5) Penetration-Aspiration Scale (PAS) scoring. All FEES examinations were performed by the same physician and video recorded to ensure assessment consistency and traceability. Twenty-one patients (55.3%) had undergone tracheostomy; all patients had nasogastric tubes *in situ*.

**Figure 1:**
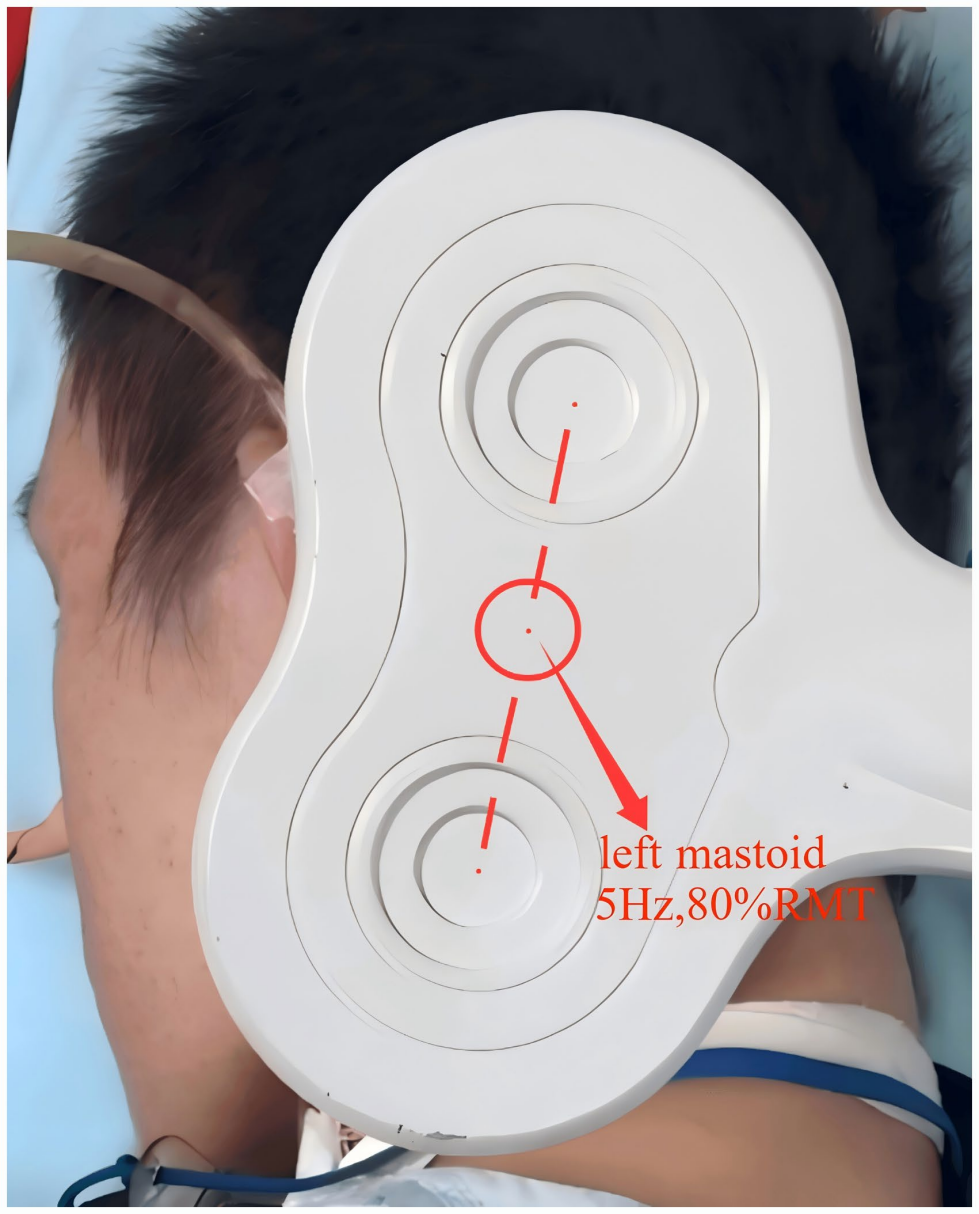
Schematic diagram of non-invasive vagus nerve magnetic stimulation treatment.

For patients receiving non-invasive vagus nerve magnetic stimulation combined with basic training, the treatment protocol was as follows. All patients received a comprehensive 21-day treatment regimen, including nVNMS and basic training components, with both treatments initiated on day 1 and concluded on day 21. Magnetic stimulation treatment used the YRD CCY-II magnetic stimulator manufactured by Yiruide Medical (Yiruide Medical Technology Co., Ltd., China). To ensure patient safety and prevent the risk of cardiac complications, the vagus nerve was stimulated on the left side, with the magnetic stimulation coil placed in the left mastoid region. Patients were positioned in a lateral recumbent position with the head slightly turned to expose the left mastoid. Stimulation parameters were set as: frequency 5 Hz, stimulation time 6 s, interval time 24 s, stimulation intensity 80% resting motor threshold, total pulse count 1,200, single treatment duration 20 min, once daily for 21 consecutive days. Treatment was implemented by a fixed medical team to ensure consistency of parameter settings and operational procedures. Each treatment session was documented in detail, recording stimulation parameters, patient tolerance, and immediate responses (see Figure 1).

Basic training included three components, with all patients receiving 21 consecutive days of basic training conducted synchronously with nVNMS treatment. Low-frequency pulsed electrical stimulation for swallowing function was administered using a VitalStim therapeutic device (Chattanooga, USA). One pair of self-adhesive electrodes was positioned as follows: one electrode was placed on the suprahyoid muscle group on one side, and the other electrode was placed on the suprahyoid muscle group on the contralateral side. The frequency was set at 80 Hz, and the intensity was adjusted according to the patient’s tolerance (usually 4–10 mA) to elicit visible contraction of the swallowing muscles without causing discomfort. Each treatment session lasted 20 min, once daily, for 21 consecutive days. Swallowing manual training was guided by rehabilitation therapists, with treatment protocols selected based on patient conditions, mainly including oral sensory stimulation, oral motor exercises, Mendelsohn maneuver, Masako maneuver, feeding training, etc., once daily, 30 min per session, for 15 consecutive days. Pulmonary rehabilitation manual training for tracheostomy patients included airway clearance, breathing exercises, and tube occlusion training selected based on specific patient conditions, once daily, 30 min per session, for 15 consecutive days. All patients (100%) completed the full 21-day course of nVNMS and basic training, with no treatment interruptions due to compliance or tolerance issues.

Pre- and post-treatment assessments were performed by evaluators who were independent of the treatment team. To minimize assessment bias, evaluators were instructed to score each assessment based solely on the patient’s performance during that session, without reference to or knowledge of the scores from other time points; assessment records from different time points were stored separately and were not accessible to the evaluator at the time of any given assessment. It should be acknowledged that in a pre-post single-arm design without sham control, complete blinding of evaluators to treatment status is not feasible, as patients’ clinical status visibly changes over the course of treatment. The blinding procedure described above was therefore implemented to minimize, but cannot fully eliminate, potential assessment bias due to evaluator awareness of the treatment trajectory. The primary evaluator was a swallowing specialist physician with over 20 years of clinical experience, responsible for laryngoscopy and Penetration-Aspiration Scale scoring; swallowing function scale assessments were completed by rehabilitation therapists who received standardized training. Assessment time points were baseline period (day 1 before treatment), end of treatment period (day 21), and a short-term follow-up period (day 28 after treatment). The 28-day follow-up was selected based on the constraints of the inpatient clinical setting and was designed to provide preliminary information on immediate post-treatment functional maintenance; it was not intended to evaluate long-term functional durability or neuroplasticity-mediated recovery, which will require extended follow-up in future controlled trials. Primary outcome measures included: Standardized Swallowing Assessment (total score 18–46 points, with higher scores indicating poorer function), Food Intake Level Scale (graded 1–10, with higher grades indicating better oral feeding ability), Penetration-Aspiration Scale (graded 1–8, assessing swallowing safety), tracheostomy cannula removal rate, and nasogastric tube removal rate. Secondary outcome measures included: vocal cord mobility (assessed bilaterally for adduction and abduction function by laryngoscopy, graded as normal, reduced, or fixed), vocal cord paralysis incidence, cough reflex assessment, cough effectiveness (graded as completely effective, effective, slightly effective, or ineffective), Murray secretion score (assessing oropharyngeal secretion accumulation, scored 0–3), swallowing frequency (recording number of spontaneous swallows per minute, with ≥3/min as normal, <3/min as reduced, and no spontaneous swallowing as absent), and adverse events (recording all adverse events occurring during treatment, including local pain, dizziness, nausea, arrhythmia, etc.). Cough reflex assessment method: The cough reflex was elicited and evaluated during fiberoptic endoscopic evaluation of swallowing (FEES) examination by the same swallowing specialist physician with over 20 years of experience using a standardized mechanical stimulation method. The specific operational procedure was as follows: (1) Stimulation method: gentle contact stimulation using the tip of a fiberoptic laryngoscope (Pentax VNL-1170 K, outer diameter 3.4 mm); (2) Stimulation site: posterior pharyngeal wall at the oropharyngeal level; (3) Stimulation process: light contact of the endoscope tip with the posterior pharyngeal wall for 1–2 s; (4) Number of stimulations: 3 stimulation attempts with 10-s intervals between each to allow recovery and minimize habituation effects; (5) Assessment timing: performed before any food/liquid swallowing trials in the FEES examination to avoid confounding effects of secretion accumulation or fatigue. Grading criteria were determined based on the latency and presence of cough response after mechanical stimulation, taking the best response from the 3 stimulations: Grade 1 (normal) = immediate cough response within 1 s after stimulation; Grade 2 (delayed) = delayed cough response within 1–3 s after stimulation; Grade 3 (no reflex) = no cough response within 3 s after stimulation or throughout the entire stimulation period. All FEES examinations (including cough reflex assessment) were video recorded for documentation and quality assurance. Assessments were performed by the same physician to ensure inter-assessment consistency.

Data analysis was performed using SPSS 26.0 software (IBM Corporation, Armonk, NY, USA). All statistical tests were two-tailed with a significance level of *α* = 0.05. Continuous variables were first tested for normality using the Shapiro–Wilk test; normally distributed data were expressed as mean ± standard deviation, and non-normally distributed data were expressed as median (interquartile range); categorical variables were expressed as frequency (percentage). Pre- and post-treatment comparisons used paired design statistical methods: paired *t*-test with Cohen’s d calculation for normally distributed continuous variables; Wilcoxon signed-rank test with effect size *r* = Z/√n for non-normally distributed or ordinal variables; and McNemar test for binary variables. Interpretation criteria: d ≥ 0.5 indicated a moderate effect and ≥ 0.8 a large effect; *r* ≥ 0.3 indicated a moderate effect and ≥ 0.5 a large effect.

Patient outcomes were presented through case series analysis, including descriptive statistics and paired pre- and post-treatment comparisons.

## 3 Results

As detailed in the Methods section and illustrated in Figure 1 (participant flow diagram), 38 consecutive eligible patients were enrolled during the study period. All 38 patients completed the full 21-day treatment protocol and the day-28 follow-up assessment, representing a 100% retention rate with no withdrawals, dropouts, or loss to follow-up. With respect to treatment fidelity, each enrolled patient received the full prescribed dose of each intervention component: 21 sessions of nVNMS (20 min/session), 21 sessions of swallowing electrical stimulation (20 min/session), and 15 sessions of manual swallowing training (30 min/session). The 21 tracheostomized patients additionally received 15 sessions of pulmonary rehabilitation training (30 min/session). Three patients (7.9%) required a temporary reduction in stimulation intensity during nVNMS sessions due to mild local discomfort, but all subsequently completed the full treatment course without session interruptions. The study cohort was predominantly male (29 cases, 76.3%), with a mean age of 52.16 ± 9.20 years. The median time from injury to enrollment was 98.5 (32.8–178.0) days. The wide range of this interval indicates considerable heterogeneity in recovery stage at enrollment, spanning from early subacute to chronic phases. While the majority of participants were likely in the subacute-to-chronic transition based on the median value, patients enrolled at the lower end of this range may still have been within an active spontaneous neurological recovery window, which should be considered when interpreting the observed outcomes.

Regarding etiological characteristics, cerebral hemorrhage was the primary cause of brain injury (22 cases, 57.9%), followed by cerebral infarction (8 cases, 21.1%), traumatic brain injury (6 cases, 15.8%), and other etiologies (2 cases, 5.3%). Neuroanatomical analysis revealed a predominance of infratentorial lesions (21 cases, 55.3%), a distribution pattern consistent with the established association between brainstem lesions and combined vocal cord-swallowing dysfunction. Supratentorial lesions were present in 12 patients (31.6%), and mixed-site lesions affected 5 patients (13.2%). For the 6 traumatic brain injury patients, lesion distribution was: 2 cases with supratentorial lesions (both with bilateral frontal lobe contusions involving the insula-frontal operculum, suggesting bilateral corticobulbar tract injury), 1 case with infratentorial lesion (isolated pontomedullary junction contusion directly involving the swallowing center and vagus nerve nuclei), and 3 cases with mixed lesions (diffuse axonal injury or multiple contusions involving both supratentorial and infratentorial structures). Among traumatic brain injury patients, 50% had mixed lesions (3/6), higher than the overall population’s mixed lesion proportion (13.2%), reflecting the complexity and multifocal nature of traumatic brain injury patterns.

The patient population exhibited considerable heterogeneity in baseline consciousness levels: 22 patients (57.9%) were fully conscious, 8 patients (21.1%) were in a minimally conscious state, and another 8 patients (21.1%) remained in a comatose state. This diversity in consciousness states is clinically relevant, as consciousness level may influence treatment response and rehabilitation potential. Due to the small subgroup sizes resulting from this three-dimensional heterogeneity across etiology, lesion location, and consciousness level, formal between-subgroup statistical analyses were not performed, as they would lack adequate power and risk inflating Type I error. The potential impact of this heterogeneity on the observed outcomes is discussed in Section 4.1.4.

The comorbidity burden was substantial, with hypertension being the most common comorbidity (22 cases, 57.9%), followed by hyperlipidemia (12 cases, 31.6%) and diabetes (5 cases, 13.2%), with other medical comorbidities present in 19 patients (50.0%). Notably, aspiration pneumonia was prevalent at baseline (34 cases, 89.5%), highlighting the severe impairment of airway protection mechanisms in this patient population.

Laryngoscopy revealed unilateral vocal cord paralysis in 6 cases (15.8%) and bilateral vocal cord paralysis in 32 cases (84.2%). The severity of swallowing dysfunction necessitated extensive supportive interventions: all patients (100%) required nasogastric tube feeding at baseline, and more than half (21 cases, 55.3%) had undergone tracheostomy for airway management. These indicators collectively demonstrate the severe functional impairment and medical complexity of this patient cohort (see Table 1).

**Table 1:** Baseline characteristics of patients with vocal cord paralysis and dysphagia following brain injure.

Characteristic	Total (*n* = 38)
Demographic characteristics	
Age (years), mean ± SD	52.16 ± 9.20
Sex, *n* (%)	
Male	29 (76.3)
Female	9 (23.7)
Disease characteristics	
Disease duration (days), median (IQR)	98.5 (32.8–178.0)
Disease type, *n* (%)	
Cerebral hemorrhage	22 (57.9)
Cerebral infarction	8 (21.1)
Traumatic brain injury	6 (15.8)
Other	2 (5.3)
Lesion location, *n* (%)	
Supratentorial	12 (31.6)
Infratentorial	21 (55.3)
Mixed	5 (13.2)
Vocal cord paralysis, *n* (%)	
Unilateral	6 (15.8)
Bilateral	32 (84.2)
Consciousness level, *n* (%)	
Alert	22 (57.9)
Minimally conscious	8 (21.1)
Comatose	8 (21.1)
Comorbidities, *n* (%)	
Hypertension	22 (57.9)
Diabetes	5 (13.2)
Hyperlipidemia	12 (31.6)
Other comorbidities	19 (50.0)
Complications, *n* (%)	
Pneumonia	34 (89.5)
Tube placement status, *n* (%)	
Tracheostomy cannula in situ	21 (55.3)
Nasogastric tube *in situ*	38 (100.0)

Continuous variables with normal distribution are expressed as mean ± SD, skewed distributions as median (IQR), and categorical variables as frequency (percentage). Lesion location was determined according to the “culprit lesion” principle, whereby the lesion site most likely to cause vocal cord paralysis and dysphagia was classified based on neuroanatomical pathway analysis and clinical relevance. Supratentorial lesions: lesions located entirely above the tentorium cerebelli; Infratentorial lesions: lesions located entirely below the tentorium cerebelli (including brainstem and cerebellum); Mixed lesions: lesions involving both supratentorial and infratentorial regions. For traumatic brain injury patients, classification was based on the primary injury site affecting lower cranial nerve pathways, with diffuse axonal injury or multiple contusions involving both supratentorial and infratentorial structures classified as mixed lesions.

SD, standard deviation; IQR, interquartile range.

To provide preliminary descriptive information on whether patients with differing baseline severity levels showed different functional outcomes, we present exploratory subgroup descriptions stratified by two objective severity indicators. These analyses are strictly descriptive and are not intended as formal hypothesis tests; subgroup sample sizes are insufficient for adequately powered statistical inference, and the results should be interpreted as hypothesis-generating only.

Among the 21 patients with tracheostomy at baseline (reflecting the most severe airway management requirement), the decannulation rate was 52.4% (11/21) by day 21, indicating that meaningful functional recovery was achievable even in this most severely impaired subgroup. Among the 17 patients without tracheostomy at baseline, nasogastric tube removal rates by day 21 were [11/17, 64.7%], and all other outcome trajectories are summarized in Table 2. Regarding vocal cord paralysis severity, among the 32 patients with bilateral vocal cord paralysis at baseline—the more severe presentation—the proportion achieving complete vocal cord recovery by day 21 was [11/32, 34.4%], compared with [3/6, 50%] among the 6 patients with unilateral paralysis. These descriptive patterns suggest that baseline severity may influence the degree of functional recovery, though formal subgroup analyses with adequate statistical power are required to test this hypothesis and will be incorporated as pre-specified analyses in future randomized controlled trials.

**Table 2:** Summary of clinical outcomes at Day 21 stratified by baseline tracheostomy status and vocal cord paralysis laterality.

Subgroup	*N*	Primary outcome	*n* (%)
By baseline tracheostomy status			
Tracheostomized	21	Decannulation at Day 21	11 (52.4%)
Non-tracheostomized	17	Nasogastric tube removal at Day 21	11 (64.7%)
By vocal cord paralysis laterality			
Bilateral paralysis	32	Complete vocal cord recovery at Day 21	11 (34.4%)
Unilateral paralysis	6	Complete vocal cord recovery at Day 21	3 (50.0%)

Subgroup comparisons are descriptive only and were not powered for formal inferential analysis. Formal subgroup analyses with adequate statistical power are planned as pre-specified analyses in a future randomized controlled trial. The tracheostomized subgroup received additional pulmonary rehabilitation training (30 min/session × 21 sessions) beyond the standard protocol, which should be considered when interpreting between-subgroup differences.

After 21 days of treatment, significant improvements were observed across multiple functional dimensions. In terms of swallowing function, SSA scores decreased from 36.92 ± 4.71 to 32.63 ± 7.10 (*p* < 0.001), and the median Food Intake Level Scale (FILS) score increased from 2 (2–2) to 3 (2–3) (*p* < 0.001). Detailed distribution changes in FILS scores showed: at baseline, all 38 patients (100%) were completely tube-feeding dependent (FILS grades 1–3), with 84.2% (32 cases) at grade 2 and 15.8% (6 cases) at grade 3, with no patients achieving oral feeding level (≥grade 4); by day 21 post-treatment, 68.4% (26 cases) remained at grade 2, 26.3% (10 cases) progressed to grade 3, and 5.3% (2 cases) transitioned to grade 4 (beginning small amounts of oral feeding). Although both grade 2 and grade 3 fall within the ‘severe dysphagia’ category of the FILS, this transition carries important clinical significance. Grade 2 patients experience severe aspiration with all swallowing attempts and are suitable only for indirect swallowing training. In contrast, grade 3 patients have reduced aspiration risk under optimal conditions and are ready to begin small-volume direct feeding training—a critical prerequisite for transitioning to oral nutrition. This grade transition therefore reflects meaningful improvements in airway protection function, swallowing reflex sensitivity, and swallowing-respiration coordination. Notably, 12 patients (31.6%) achieved FILS ≥ grade 6 (able to obtain nutrition orally for all three meals), as evidenced by successful nasogastric tube removal, indicating that the distribution of FILS improvements extended beyond the severe range in a clinically meaningful subset. The FILS improvements should be interpreted in conjunction with the concurrent PAS reduction (grade 6 → 4) and SSA score decrease (36.92 → 32.63), which together reflect convergent functional gains across multiple dimensions of swallowing safety and capacity. Swallowing safety indicators showed that PAS scores decreased from baseline 6 (4–6) to 4 (4–4) (*p* < 0.001), representing clinically significant enhancement of airway protection function. Secondary swallowing parameters also showed improvement: Murray secretion scores decreased from 3 (1–3) to 2 (1–2) (*p* = 0.006), and the distribution of swallowing frequency scores showed a statistically significant change, with the interquartile range narrowing from (2–2) to (1–2) (*p* = 0.001), indicating reduced variability toward higher swallowing frequency.

Vocal cord function assessment revealed significant improvement in vocal cord mobility post-treatment. Laryngoscopy showed that the incidence of vocal cord paralysis decreased from 38 cases (100%) at baseline to 24 cases (63.2%) post-treatment (*p* < 0.001). Left vocal cord adduction scores decreased from 2 (2–2) to 1 (1–1) (*p* < 0.001), and right vocal cord adduction improved from 2 (1–2) to 1 (1–1) (*p* = 0.002). Vocal cord abduction function also showed improvement: left vocal cord abduction scores improved from 1 (1–1) to 1 (1–1) (*p* = 0.021), and right vocal cord abduction improved from 2 (2–2) to 2 (1–2) (*p* = 0.019). These improvements in vocal cord function have important clinical implications for phonation and laryngeal vestibule closure during swallowing.

Consistent with physiological function improvements, airway protection mechanisms were significantly enhanced. Cough reflex assessed by standardized mechanical stimulation of the posterior pharyngeal wall improved from 2 (2–2) to 2 (1–2) (*p* = 0.007), and cough effectiveness improved from 2 (1–3) to 2 (2–2) (*p* = 0.005), indicating enhanced airway clearance capacity of coughing. These functional improvements translated into significant reductions in medical device dependence: Tracheostomy cannula retention rate decreased from 21 cases (55.3%) to 10 cases (26.3%) post-treatment (*p* = 0.001), and nasogastric tube dependence decreased from 38 cases (100%) to 26 cases (68.4%) (*p* < 0.001). For the 11 patients who achieved decannulation, liberation from tracheostomy management reduced infection risk and, in many cases, partially restored phonation and effective coughing. For the 12 patients who achieved nasogastric tube removal, the ability to eat orally restored a basic dimension of daily social life and reduced caregiver burden associated with tube management (see Table 3).

**Table 3:** Comparison of clinical outcomes before and after vagus nerve magnetic stimulation treatment (*n* = 38).

Parameter	Pre-treatment	Post-treatment	Test statistic	Effect size	*p* value	Statistical test
Swallowing function						
SSA score, mean ± SD	36.92 ± 4.71	32.63 ± 7.10	*t* = 5.435	Cohen’s d = 0.72 (large)	<0.001	Paired *t*-test
FILS score, median (IQR)	2 (2–2)	3 (2–3)	*Z* = −4.062	—	<0.001	Wilcoxon signed-rank test
Murray score,median (IQR)	3 (1–3)	2 (1–2)	*Z* = −2.725	*r* = 0.44 (moderate)	0.006	Wilcoxon signed-rank test
Swallowing frequency, median (IQR)	2 (2–2)	2 (1–2)	*Z* = −3.260	*r* = 0.53 (moderate)	0.001	Wilcoxon signed-rank test
PAS score, median (IQR)	6 (4–6)	4 (4–4)	*Z* = −3.945	*r* = 0.64 (large)	<0.001	Wilcoxon signed-rank test
Vocal cord examination findings						
Left vocal cord adduction, median (IQR)	2 (2–2)	1 (1–1)	*Z* = −4.092	*r* = 0.66 (large)	<0.001	Wilcoxon signed-rank test
Right vocal cord adduction, median (IQR)	2 (1–2)	1 (1–1)	*Z* = −3.128	*r* = 0.51 (moderate)	0.002	Wilcoxon signed-rank test
Left vocal cord abduction, median (IQR)	1 (1–1)	1 (1–1)	*Z* = −2.310	*r* = 0.37 (small)	0.021	Wilcoxon signed-rank test
Right vocal cord abduction, median (IQR)	2 (2–2)	2 (1–2)	*Z* = −2.352	*r* = 0.38 (small)	0.019	Wilcoxon signed-rank test
Vocal cord paralysis, *n* (%)	38 (100.0)	24 (63.2)	*χ*^2^ = 12.071	—	<0.001	McNemar test
Airway protection function						
Cough reflex, median (IQR)	2 (2–2)	2 (1–2)	*Z* = −2.714	*r* = 0.44 (moderate)	0.007	Wilcoxon signed-rank test
Cough effectiveness, median (IQR)	2 (1–3)	2 (2–2)	*Z* = −2.840	*r* = 0.46 (moderate)	0.005	Wilcoxon signed-rank test
Tube placement status						
Tracheostomy cannula *in situ*, *n* (%)	21 (55.3)	10 (26.3)	*χ*^2^ = 9.091	—	0.001	McNemar test
Nasogastric tube *in situ*, *n* (%)	38 (100.0)	26 (68.4)	*χ*^2^ = 10.083	—	<0.001	McNemar test

Continuous variables are expressed as mean ± SD or median (IQR); categorical variables are expressed as frequency (percentage). Paired *t*-test was used for normally distributed continuous variables; Wilcoxon signed-rank test was used for ordinal variables and non-normally distributed continuous variables; McNemar test was used for paired categorical variables. *p* < 0.05 indicates statistical significance.

SSA, standardized swallowing assessment (total score 18–46 points, higher scores indicate poorer function); FILS, food intake level scale (Fujishima scale, graded 1–10, higher grades indicate better oral feeding ability; key clinical boundaries: grades 1–3 = complete tube-feeding dependence [severe, no oral nutrition], grade 2 patients suitable only for indirect swallowing training, grade 3 patients ready to begin direct feeding training; grades 4–6 = mixed oral-tube feeding [moderate]; grades 7–10 = complete oral nutrition [mild to normal, no tube feeding required]); PAS, Penetration-Aspiration Scale (graded 1–8, higher grades indicate greater aspiration risk); Murray secretion score (0–3 points, higher scores indicate more severe oropharyngeal secretion accumulation); swallowing frequency (1 = ≥3 times/min, 2 = <3 times/min, 3 = no spontaneous swallowing); vocal cord function (1 = normal, 2 = reduced, 3 = fixed); cough reflex (1 = normal [immediate cough within 1 s after stimulation], 2 = delayed [cough within 1–3 s after stimulation], 3 = no reflex [no cough response within 3 s after stimulation or throughout entire stimulation period]; best response of 3 stimulation attempts recorded); cough effectiveness (1 = completely effective, 2 = effective, 3 = slightly effective, 4 = ineffective); IQR, interquartile range; SD, standard deviation.

Short-term follow-up at day 28 showed that the functional improvements observed at day 21 were maintained within the observation window, with tracheostomy cannula retention rate at 18.4% (7 cases) and nasogastric tube retention rate at 57.9% (22 cases). It should be noted that the continued modest increase in tube removal rates between day 21 and day 28 suggests that functional recovery may not have reached a plateau at the end of the observation period; however, whether these improvements were sustained beyond day 28 cannot be determined from the present data (see Table 4).

**Table 4:** Changes in tube placement status post-treatment (*n* = 38).

Follow-up indicator	Day 21 post-treatment	Day 28 post-treatment	Change	Trend
Tracheostomy cannula placement status				
Number with tube in situ	10	7	−3	↓
Retention rate (%)	26.3	18.4	−7.9 percentage points	↓
Nasogastric tube placement status				
Number with tube in situ	26	22	−4	↓
Retention rate (%)	68.4	57.9	−10.5 percentage points	↓

↓ indicates a decrease in retention rate, suggesting an increase in tube removal rate. No tube-related complications occurred during the follow-up period.

Throughout the 21-day treatment period, all participants tolerated the intervention protocol and completed the prescribed treatment course. Three patients (7.9%) reported mild discomfort at the stimulation site, which resolved after reducing stimulation intensity without affecting treatment continuation. No moderate or severe adverse events were recorded, and no arrhythmias, seizures, or other complications requiring medical intervention occurred. Safety monitoring conducted during treatment included assessment of vital signs (blood pressure, heart rate, oxygen saturation) before each treatment session, with no abnormalities detected (see Table 5). This favorable safety profile is consistent with the non-invasive nature of the intervention and supports the feasibility and clinical application prospects of this treatment approach in this medically complex patient population.

**Table 5:** Safety data during treatment period (*n* = 38).

Safety indicator	Result
Treatment completion status	
Completed full 21-day course	38 (100%)
Treatment interrupted due to adverse events	0 (0%)
Adverse events	
Mild discomfort (discomfort at stimulation site)	3 (7.9%)
Management	Symptoms resolved after reducing stimulation intensity
Impact on treatment	No impact on treatment continuation
Moderate adverse events	0 (0%)
Severe adverse events	0 (0%)
Specific complications	
Arrhythmia	0 (0%)
Seizure	0 (0%)
Other complications requiring medical intervention	0 (0%)
Vital signs monitoring	
Abnormal blood pressure	0 (0%)
Abnormal heart rate	0 (0%)
Abnormal oxygen saturation	0 (0%)

Vital signs were assessed before each treatment session. All participants tolerated the intervention protocol.

## 4 Discussion

This prospective case series study represents, to our knowledge, one of the earliest systematic clinical evaluations of the clinical efficacy and safety of non-invasive vagus nerve magnetic stimulation (nVNMS) in patients with vocal cord paralysis and dysphagia following brain injury. After 21 days of the combined intervention protocol, significant improvements were observed across multiple functional domains. Swallowing function improved: SSA scores decreased by 11.6% (from 36.92 ± 4.71 to 32.63 ± 7.10, *p* < 0.001), and median FILS scores improved by one grade (from grade 2 to grade 3, *p* < 0.001). Airway protection also improved, with PAS scores decreasing by two grades (from grade 6 to grade 4, *p* < 0.001). Vocal cord paralysis incidence decreased from 100% to 63.2% (*p* < 0.001). Device dependence was reduced: tracheostomy cannula retention rate decreased from 55.3 to 26.3% (*p* = 0.001), and nasogastric tube retention rate decreased from 100% to 68.4% (*p* < 0.001).

The clinical relevance of these changes warrants distinction between outcome measures with direct impact on patient care status and those reflecting underlying physiological changes. The decannulation rate (52.4%, 11/21 tracheostomy patients) and nasogastric tube removal rate (31.6%, 12/38 patients) represent concrete transitions from device-dependent to device-free management, with direct implications for complication risk reduction and quality of life. Among physiological indicators, the PAS reduction from grade 6 to grade 4 represents a threshold crossing from absent to present airway protective response: PAS grade 6 denotes material entering the airway below the vocal folds without ejection, reflecting complete failure of airway protection; PAS grade 4 denotes material contacting the vocal folds and being successfully expelled, reflecting restoration of functional airway protection. The SSA reduction of 4.29 points reflects improvement across oral preparation, pharyngeal transit, and laryngeal protection phases. The clinical significance of each outcome measure is discussed in detail in Section 4.3.

These results suggest that the multimodal protocol incorporating nVNMS alongside swallowing electrical stimulation and manual training may be associated with clinically relevant functional improvements in this patient population. However, given the absence of a control group and the simultaneous delivery of multiple active interventions, the specific contribution of nVNMS cannot be isolated from those of co-administered therapies or natural recovery.

### 4.1 Comparison of study findings with existing literature

#### 4.1.1 Swallowing function improvement

This study observed an 11.6% improvement in SSA scores. Conventional electrical stimulation and exercise alone yield moderate improvements in swallowing scores (7–9, 29). By contrast, the combined protocol employed in our study, which included nVNMS alongside standard swallowing rehabilitation, was associated with observable functional gains even in this severely impaired population. Previous randomized controlled studies have shown that transcutaneous auricular vagus nerve stimulation (taVNS) significantly improved swallowing function scores after 4 weeks of treatment in post-stroke dysphagia patients, with the treatment group showing significantly shortened swallowing reflex latency compared to the control group (14). Other studies have confirmed that transcranial direct current stimulation of the vagus nerve can significantly reduce Standardized Swallowing Scale scores and Functional Dysphagia Scale scores in post-stroke dysphagia patients while decreasing serum inflammatory factors IL-1β and IL-8 levels, suggesting that vagus nerve modulation may improve swallowing function by suppressing inflammatory responses (30).

A randomized double-blind controlled trial further confirmed that after 4 weeks of transcranial direct current stimulation of the vagus nerve combined with conventional swallowing rehabilitation, the treatment group showed significantly greater improvements in Fiberoptic Endoscopic Evaluation of Swallowing Severity Score, Modified Mann Assessment of Swallowing Ability, Functional Communication Measures, and PAS scores compared to the control group (31).

Notably, the patient population in this study had more severe functional impairments: 84.2% had bilateral vocal cord paralysis, 89.5% had aspiration pneumonia, 55.3% required tracheostomy, and the median disease duration was 98.5 days (subacute to chronic phase), whereas previous studies mostly enrolled patients with isolated dysphagia without tracheostomy. Despite worse baseline function, improvements were still observed following the combined intervention protocol in this study, suggesting that this multimodal approach may warrant further investigation in patients with severe functional impairment. Of note, the PAS improvement observed in this study (grade 6 → 4, a two-grade reduction) is directionally consistent with PAS improvements reported by Chen et al. (31) in their randomized controlled trial of transcranial direct current stimulation of the vagus nerve combined with conventional swallowing rehabilitation, where the treatment group showed significantly greater PAS score reductions compared to the control group, suggesting that vagus nerve modulation may confer airway protection benefits across different stimulation modalities. It should be noted, however, that the wide enrollment range (32.8–178.0 days) introduces heterogeneity in spontaneous recovery potential across the cohort, which represents an additional limitation when interpreting the observed improvements and must be accounted for in future controlled studies.

#### 4.1.2 Vocal cord function recovery

Regarding vocal cord function, this study observed complete recovery from vocal cord paralysis in 36.8% of patients (14/38), with 63.2% of patients still having vocal cord paralysis post-treatment. Direct literature on nVNMS treatment for vocal cord paralysis is currently limited. Previous studies have shown that unilateral vocal cord paralysis patients who underwent arytenoid adduction combined with laryngoplasty not only improved phonation function but also showed significant improvements in Eating Assessment Tool-10 (EAT-10) scores, liquid aspiration, and peak cough flow, indicating that adequate glottic closure plays an important role in swallowing function (32). A study of patients with recurrent laryngeal nerve paralysis after esophagectomy found that patients with recurrent laryngeal nerve paralysis combined with dysphagia had significantly reduced hyoid elevation amplitude (10.62 mm vs. 16.75 mm, *p* = 0.003), suggesting that compensatory hyoid elevation to enhance epiglottic retroversion is crucial for patients with incomplete glottic closure (33). The observation in this study that some patients improved but did not fully recover vocal cord mobility may be related to lesion location (55.3% infratentorial lesions), injury severity, and disease duration. Infratentorial lesions—particularly those involving the brainstem—may directly damage the nucleus ambiguus and vagal motor fibers innervating the intrinsic laryngeal muscles, inherently limiting vocal cord recovery potential regardless of intervention. In contrast, supratentorial lesions primarily impair corticobulbar projections while leaving brainstem vagal circuitry relatively intact, potentially conferring a more favorable recovery trajectory. This neuroanatomical distinction likely contributes to the outcome variance observed across the cohort.

#### 4.1.3 Reduction in medical device dependence

This study observed successful decannulation in 52.4% of tracheostomy patients (11/21) and successful nasogastric tube removal in 31.6% of patients (12/38). A study on preservation of the internal branch of the superior laryngeal nerve (ibSLN) during transoral endoscopic surgery for hypopharyngeal squamous cell carcinoma found that patients in the ibSLN preservation group had faster postoperative swallowing function recovery, with nasogastric tube removal occurring 6 days earlier than the control group, and MD Anderson Dysphagia Inventory scores at 14 days were significantly better than the control group in overall function, functional dimension, and physical dimension (34). These results echo the reduction in medical device dependence in this study, suggesting that protection or recovery of neural function is a key factor in improving swallowing function and reducing tube feeding and airway management needs. However, due to the lack of a control group in this study, the contribution of natural recovery cannot be completely excluded, which is an important limitation of the study.

#### 4.1.4 Potential impact of cohort heterogeneity on findings

The cohort’s heterogeneity across etiology, lesion location, and consciousness level warrants specific discussion.

Lesion location: Infratentorial lesions (55.3%) may directly damage the vagal motor nuclei (nucleus ambiguus, nucleus tractus solitarius)—the theoretical central targets of nVNMS—potentially attenuating the treatment response in this subgroup compared with supratentorial lesion patients, in whom brainstem circuitry remains relatively intact. This mechanistic distinction may partly explain why only 36.8% of patients achieved complete vocal cord recovery. Future studies should stratify analyses by lesion location to test this hypothesis.

Consciousness level: Patients with disorders of consciousness (42.2%) could not perform volitional swallowing maneuvers, meaning the “combined intervention” was functionally non-equivalent across subgroups: conscious patients received the full multimodal protocol, while unconscious patients received only passive stimulation (nVNMS and electrical stimulation). Improvements in the unconscious subgroup may therefore reflect passive stimulation effects and spontaneous recovery rather than the full rehabilitative protocol.

Etiology: Cerebral hemorrhage (57.9%), ischemic stroke (21.1%), and TBI (15.8%) differ in natural recovery trajectories and neuroplasticity potential. The predominantly hemorrhagic cohort may limit generalizability to ischemic or TBI populations. These three dimensions of heterogeneity represent important uncontrolled sources of variance; adequately powered RCTs with pre-specified subgroup analyses are required to determine whether treatment response differs across these patient characteristics.

Baseline functional severity: Beyond etiology, lesion location, and consciousness level, baseline functional severity itself represents a fourth dimension of heterogeneity with potential to substantially influence treatment outcomes. In the present cohort, baseline severity was uniformly high—100% tube feeding dependence, 55.3% tracheostomy, 84.2% bilateral vocal cord paralysis, and 89.5% aspiration pneumonia prevalence—yet within this severely impaired population, meaningful within-group variation in severity existed (e.g., tracheostomized vs. non-tracheostomized; bilateral vs. unilateral vocal cord paralysis). Patients with more severe baseline impairment may have a greater ceiling for measurable improvement but a lower neurobiological reserve for functional recovery; conversely, patients with less severe impairment may have a higher recovery ceiling but smaller absolute gains on device-dependence measures. Exploratory descriptive data stratified by tracheostomy status and vocal cord paralysis laterality are provided in Section 3 above. Formal examination of whether baseline severity moderates treatment response will require adequately powered randomized controlled trials with baseline severity as a stratification variable and pre-specified subgroup analyses.

### 4.2 Potential mechanisms of action

The potential mechanisms by which nVNMS improves swallowing and vocal cord function involve multi-level neuromodulatory effects including anatomical foundation, peripheral nerve pathway activation, central nervous regulation, and neuroplasticity.

#### 4.2.1 Anatomical foundation and neural innervation of Pharyngolaryngeal muscles

In terms of anatomical foundation, the pharyngeal plexus is a key neural network for swallowing movement, composed of the pharyngeal branch of the vagus nerve, glossopharyngeal nerve, and cervical sympathetic nerve fibers (35). Anatomical studies have described the structure of the pharyngeal plexus in detail, finding that it is primarily formed by the pharyngeal branch of the vagus nerve and innervates the motor and sensory functions of the pharyngeal constrictor muscles, which is important for understanding postoperative dysphagia and developing protective strategies (36).

The vagus nerve exerts precise control over pharyngolaryngeal swallowing muscles and vocal cords through its branches. The superior laryngeal nerve, as the first branch of the vagus nerve, provides sensory innervation to the mucosa of the vallecula and supraglottic region through its internal branch, monitoring bolus position and triggering the swallowing reflex; its external branch provides motor innervation to the cricothyroid muscle, regulating vocal cord tension. The recurrent laryngeal nerve, another important branch of the vagus nerve, provides motor innervation to all intrinsic laryngeal muscles except the cricothyroid muscle, including the thyroarytenoid muscle (vocal cord adduction), posterior cricoarytenoid muscle (vocal cord abduction), lateral cricoarytenoid muscle and interarytenoid muscles (glottic closure), as well as the lower pharyngeal constrictor muscles and cricopharyngeus muscle involved in swallowing, while providing sensory input to the subglottic mucosa (20).

nVNMS is positioned to target the proximal left vagus nerve at the mastoid region and is theoretically expected to activate both the superior laryngeal nerve and recurrent laryngeal nerve, potentially forming multiple regulatory effects on pharyngolaryngeal muscles: First, it may enhance cricothyroid muscle function through the external branch of the superior laryngeal nerve, improving vocal cord tension and voice quality; Second, it may activate intrinsic laryngeal muscles (thyroarytenoid, cricoarytenoid muscles, etc.) through the recurrent laryngeal nerve, improving glottic closure and airway protection; Third, it may regulate pharyngeal constrictor muscles and palatopharyngeal muscles through the pharyngeal branch, strengthening pharyngeal propulsion. This theorized simultaneous activation of swallowing muscles and vocal cord muscles may provide a plausible anatomical rationale for concurrent functional improvements, though no electrophysiological data were collected in the present study to confirm that this activation actually occurred. This mechanism is most applicable in patients with preserved brainstem circuitry (supratentorial lesions); patients with direct infratentorial brainstem damage involving the vagal nuclei may experience a fundamentally different or more limited response through this pathway.

#### 4.2.2 Enhanced Vagus nerve-mediated sensory afferents

nVNMS enhances the sensory afferent pathway of pharyngolaryngeal swallowing muscles by stimulating the vagus nerve trunk, thereby synchronously activating its important branch—the superior laryngeal nerve. The internal branch of the superior laryngeal nerve has densely distributed receptors in the vallecula, arytenoid cartilage, and pharyngeal mucosa, monitoring the mechanical stimulation and chemical properties of the bolus; this sensory information is a key triggering factor for initiating and regulating the swallowing reflex (23, 32).

Studies in piglet models found that after superior laryngeal nerve injury, swallowing duration was shortened and hyoid movement distance decreased, reflecting decreased coordination of pharyngolaryngeal muscles, though different individuals showed variable responses to nerve injury, possibly related to individual differences in neural connectivity (37). Electrophysiological studies found that continuous electrical stimulation of the superior laryngeal nerve in anesthetized rats could inhibit the initiation of the swallowing reflex, suggesting that sensory input from the superior laryngeal nerve has complex regulatory effects on the swallowing reflex (38). Further research confirmed that local application of ATP in swallowing-related peripheral areas could promote swallowing reflex triggering through P2X3 receptors, suggesting that peripheral sensory enhancement is an important mechanism for improving swallowing function (39).

Based on the neuroanatomical relationships described above, it is theoretically hypothesized that nVNMS may improve pharyngolaryngeal muscle function through the following pathways: magnetic stimulation may activate afferent fibers of the vagus nerve trunk, potentially enhancing sensory afferent input from the pharyngolaryngeal mucosa via the superior laryngeal nerve; this enhanced sensory input may, in turn, increase swallowing reflex sensitivity and promote more coordinated contraction of pharyngeal constrictors, suprahyoid muscles, and intrinsic laryngeal muscles. The present study observed improvements in Murray secretion scores (grade 3 → 2, *p* = 0.006) and swallowing frequency (*p* = 0.001), which are potentially consistent with the hypothesis that oropharyngeal sensorimotor function was enhanced. However, these clinical observations cannot be directly attributed to vagus nerve-superior laryngeal nerve pathway activation, as no electrophysiological or sensory threshold measurements were collected in this study to confirm this mechanistic link.

#### 4.2.3 Central neural pathway coordination of Pharyngolaryngeal muscles

At the central level, it is theoretically hypothesized that magnetic field activation of vagus nerve afferent fibers may transmit signals to the nucleus tractus solitarius (NTS) in the medulla. The NTS is an established component of the swallowing center that, based on neuroanatomical evidence, receives sensory information from the pharyngolarynx and coordinates reflexive contraction of swallowing-related muscles (23). Supporting the plausibility of this pathway, animal experiments have demonstrated that mechanical stimulation of the esophageal wall can activate the medullary swallowing center through vagus nerve afferent pathways (40)—though whether comparable central activation occurs following non-invasive magnetic stimulation in humans has not been directly demonstrated and was not measured in the present study.

Additionally, it has been hypothesized that nVNMS may modulate the excitability of the dorsal motor nucleus of the vagus nerve and nucleus ambiguus, potentially influencing central pattern generator (CPG) function responsible for coordinating swallowing and respiration-related muscle activities (21, 23). If such modulation were to occur, it could theoretically improve temporal coordination of pharyngeal constrictors and intrinsic laryngeal muscles. This multi-step inferential chain—from peripheral magnetic stimulation to central nucleus modulation to improved swallowing coordination—remains entirely unverified in the present study and should be regarded as a working hypothesis. Notably, prior invasive VNS research has shown that vagus nerve stimulation can elicit laryngeal motor evoked potentials (41); however, this finding was obtained using implanted electrodes rather than surface magnetic stimulation, and the extent to which it supports the central activation hypothesis for nVNMS remains uncertain.

#### 4.2.4 Neuroplasticity-mediated muscle function reconstruction

At the neuroplasticity level, repetitive vagal stimulation has been hypothesized to promote synaptic reorganization of neural pathways involved in swallowing and vocal cord control. Animal research suggests that VNS paired with rehabilitation training may drive VNS-dependent synaptic plasticity in motor networks (42), and taVNS studies in rodent cerebral ischemia models have reported findings suggestive of neuroplasticity-related processes including white matter remodeling (43). Whether analogous neuroplasticity mechanisms are engaged by non-invasive magnetic stimulation in the human clinical context studied here is unknown. The present study did not collect neurophysiological, neuroimaging, electrophysiological, or biomarker data (such as motor evoked potentials, functional MRI, or neurotrophic factor levels), and therefore no inferences regarding neuroplasticity mechanisms can be drawn from the present data. These hypotheses are noted here solely to identify directions for future mechanistic investigation. Importantly, if neuroplasticity-mediated processes are indeed engaged by nVNMS, the expected time course for such changes—typically several months for structural synaptic reorganization and white matter remodeling to manifest as stable functional gains—substantially exceeds the 28-day follow-up window of the present study. Consequently, the present data are insufficient to determine whether the observed improvements reflect durable neuroplasticity-driven reorganization or represent transient functional facilitation that may not be maintained after stimulation cessation. This temporal mismatch between the proposed neuroplasticity mechanisms and the study’s observation window is acknowledged as a fundamental limitation and is addressed further in Section 4.4.

In summary, based on the neuroanatomical properties of the vagus nerve and findings from prior VNS research across different modalities and animal models, several mechanistic pathways may theoretically be engaged by nVNMS, including peripheral nerve activation, central sensory afferent modulation, and neuroplasticity-related processes. It is essential to emphasize that all mechanistic pathways discussed in this section represent theoretical hypotheses informed by external literature—primarily from animal studies and from human studies of different VNS modalities (electrical rather than magnetic stimulation)—and none can be directly verified or attributed to nVNMS based on the data of the present study. The present study collected no neurophysiological, electrophysiological, neuroimaging, or biomarker data, and all participants simultaneously received swallowing electrical stimulation and manual training, precluding any mechanistic attribution. The mechanistic discussion above should therefore be understood solely as a framework for hypothesis generation to guide the design of future mechanistic studies, rather than as an explanation of the observed clinical improvements.

### 4.3 Clinical significance and practice implications

The results of this study may have multiple clinical implications. First, prolonged retention of tracheostomy cannulas and nasogastric tubes can lead to complications such as tracheal stenosis, nasopharyngeal injury, gastroesophageal reflux, and infections (44); the decannulation rate of 52.4% (11/21 tracheostomy patients) and nasogastric tube removal rate of 31.6% (12/38 patients) observed in this study represent concrete transitions in care status with direct implications for complication reduction, healthcare costs, and quality of life.

Second, the PAS reduction from grade 6 to grade 4 represents a qualitatively meaningful threshold crossing rather than merely a quantitative score change. PAS grade 6 denotes material entering the airway below the vocal folds with no ejection response—reflecting complete failure of airway protection and the highest aspiration risk category within the penetration range. PAS grade 4 denotes material contacting the vocal folds and being successfully expelled—reflecting restoration of a functional protective response. This two-grade improvement therefore marks the transition from absent to present airway protection, a clinically critical distinction. Considering that 89.5% of patients had already developed aspiration pneumonia at baseline, restoration of airway protective responses may help reduce pneumonia recurrence risk and shorten hospitalization (35).

Third, the SSA score reduction of 4.29 points (from 36.92 to 32.63, an 11.6% improvement) reflects gains across multiple phases of swallowing including oral preparation, pharyngeal transit, and laryngeal protection. This improvement is further corroborated by concurrent improvements in Murray secretion scores (grade 3 → 2) and swallowing frequency, which together provide convergent evidence of enhanced oropharyngeal function beyond what a single scale captures.

Fourth, partial restoration of oral feeding ability (FILS grade 2 → 3) may have positive implications for patient psychological health and social participation; oral feeding not only meets nutritional needs but is also an important component of social interaction and quality of life (45). Importantly, the 31.6% of patients who achieved nasogastric tube removal had reached FILS ≥ grade 6—indicating that functional gains were not limited to the severe dysphagia range and that a meaningful subset progressed to complete oral nutrition.

Fifth, nVNMS, as a non-invasive, safe, and easy-to-operate treatment modality with good patient tolerance (100% treatment completion) and no serious adverse events, is suitable for widespread application in rehabilitation medicine departments, especially for patients who are unwilling or unsuitable for invasive surgical treatment.

Specific recommendations for clinical practice: (1) Indication selection: nVNMS may be suitable for subacute to chronic phase patients with vocal cord paralysis and dysphagia following brain injury, especially those with severe functional impairment requiring tracheostomy and/or nasogastric tube feeding who have poor response to traditional rehabilitation training; (2) Treatment timing: The median disease duration in this study was 98.5 days; however, the enrollment range spanned from 32.8 to 178.0 days, encompassing both subacute and chronic phases. While some patients may have reached a relatively stable functional plateau, the wide range means that spontaneous neurological recovery cannot be excluded as a contributor to observed improvements in patients enrolled earlier in this range. Whether the combined protocol incorporating nVNMS may benefit patients across different phases of recovery—including those beyond the traditional “therapeutic window”—requires verification through controlled trials with stratified enrollment by injury-to-enrollment duration; (3) Combined treatment strategy: This study employed a comprehensive protocol of nVNMS combined with swallowing electrical stimulation and manual training, with all treatments conducted for 21 days synchronously; multimodal treatment may produce synergistic effects. This standardized 21-day course design ensured consistency in treatment dosage but also limited our ability to distinguish the independent efficacy of nVNMS. The efficacy of nVNMS alone requires verification through controlled trials; (4) Efficacy monitoring: It is recommended to regularly assess efficacy using standardized assessment tools (such as SSA, FILS, PAS) and objective examinations (such as laryngoscopy), and to adjust treatment plans promptly.

### 4.4 Study limitations and future directions

As a prospective case series, this study is subject to the inherent limitations of a case series design. First, the lack of a control or sham condition prevents differentiation between intervention-related improvements and spontaneous neurological recovery, which significantly affects the internal validity of this study. This issue is compounded by the wide injury-to-enrollment duration range in our sample (32.8–178.0 days). Although the median enrollment was 98.5 days post-injury—a timepoint at which the rate of spontaneous recovery in motor and swallowing function typically begins to decelerate—the lower boundary of this range means that a subset of participants may still have been within an active spontaneous recovery phase at the time of enrollment. Published literature indicates that swallowing function in patients with brainstem lesions may continue to show spontaneous improvement for up to 3–6 months post-injury, and vocal cord function recovery has been documented as late as 12 months in some cases [refs]. Consequently, it is not possible to determine from the present data whether the observed improvements reflect the effects of the combined intervention, spontaneous recovery, or their interaction. Confounding factors such as the placebo effect and Hawthorne effect also cannot be excluded. Second, the most critical limitation of this study is the simultaneous delivery of multiple active interventions to all participants, including nVNMS, low-frequency pulsed swallowing electrical stimulation, manual swallowing training, and pulmonary rehabilitation for tracheostomy patients. This multimodal design, while reflecting standard clinical practice, fundamentally prevents isolation of the specific therapeutic contribution of nVNMS from those of co-administered therapies or natural neurological recovery. Consequently, all observed improvements should be interpreted as outcomes of the combined protocol as a whole, and no causal claims regarding nVNMS can be made from these data. This limitation underscores the critical need for future randomized controlled trials with sham stimulation control groups and, if feasible, dismantling designs to determine the independent efficacy of nVNMS. Third, this was a pilot feasibility study with a sample size (*n* = 38) determined by practical recruitment feasibility rather than formal *a priori* power calculation. The enrolled sample represents all consecutive eligible inpatients during the study period and exceeds the minimum threshold of 12–30 participants recommended for pilot studies (27, 28). However, the absence of a formal a priori power calculation means that the study remains potentially underpowered to detect smaller or heterogeneous subgroup effects, and the findings should be interpreted accordingly. The large effect sizes observed (Cohen’s d = 0.72 for SSA; r = 0.64 for PAS) provide an empirical basis for sample size planning in future adequately powered randomized controlled trials, which should include stratified enrollment by etiology, lesion location, and consciousness level. Fourth, the cohort exhibits substantial heterogeneity across three dimensions. Etiological heterogeneity: the predominantly hemorrhagic composition (57.9%) may limit generalizability to ischemic stroke and TBI populations, which differ in natural recovery trajectories (46, 47). Lesion location heterogeneity: the coexistence of infratentorial (55.3%), supratentorial (31.6%), and mixed (13.2%) lesions is particularly relevant to nVNMS, as infratentorial lesions may directly damage the central vagal nuclei that are the theoretical targets of this intervention. Consciousness level heterogeneity: 42.2% of patients had disorders of consciousness at enrollment, precluding active participation in volitional swallowing training and rendering the combined intervention functionally non-equivalent across subgroups. These three dimensions collectively limit the interpretability of the aggregate findings and should be addressed through stratified RCT designs in future research. Furthermore, post-decannulation respiratory support selection—particularly between high-flow nasal cannula oxygen therapy and noninvasive ventilation, modalities shown to be clinically comparable yet to differ markedly in tolerability in high-risk extubation settings (48)—was not systematically documented in the present study, nor were outcomes compared between these modalities. This represents a limitation of the current study and a direction worthy of prospective investigation in future trials. Fifth, the 28-day follow-up period represents a significant limitation regarding the durability and long-term maintenance of the observed treatment effects. This follow-up duration was pragmatically determined by the inpatient clinical setting—patients were hospitalized at a specialized brain injury rehabilitation center with defined discharge timelines—and is consistent with follow-up durations employed in comparable pilot feasibility studies of novel neuromodulation interventions. However, this timeframe is insufficient to address several critical clinical and mechanistic questions:

(1) Durability of treatment effects: Whether the functional improvements in swallowing safety, vocal cord mobility, and reduced device dependence observed at day 21 and day 28 are maintained at clinically relevant timepoints (e.g., 3, 6, or 12 months) remains unknown. The observation that tube removal rates continued to modestly increase between day 21 and day 28 suggests that functional recovery trajectories had not yet plateaued at the end of our observation window, underscoring the need for longer follow-up to characterize the full recovery curve and identify the point of functional stabilization.
(2) Neuroplasticity-related changes: The neuroplasticity mechanisms discussed in Section 4.2.4—including VNS-dependent synaptic plasticity, cortical remapping, and white matter remodeling—are known to require weeks to months to manifest as stable, structurally consolidated functional changes. A 28-day observation window cannot capture these processes, and the present data therefore cannot confirm or refute whether neuroplasticity-mediated reorganization contributed to the observed outcomes.
(3) Late adverse events: Although no adverse events were observed during the 21-day treatment period and at the 28-day follow-up, longer observation is required to rule out delayed adverse effects that may emerge weeks or months after treatment cessation.

Future multicenter randomized controlled trials must incorporate extended follow-up periods of at minimum 3 months, and ideally 6–12 months, to characterize the full temporal profile of functional recovery, assess maintenance of treatment effects, and provide data sufficient to evaluate neuroplasticity-related mechanisms. Such trials should also include stratified randomization by etiology (hemorrhagic vs. ischemic vs. TBI), lesion location (infratentorial vs. supratentorial), consciousness level (conscious vs. disorders of consciousness), and injury-to-enrollment duration (subacute < 3 months vs. chronic ≥3 months), with pre-specified subgroup analyses to identify optimal beneficiary populations and determine whether lesion-location-specific mechanisms predict differential treatment responses. Additionally, conducting mechanistic studies using multimodal technologies such as functional MRI, transcranial magnetic stimulation, and electromyography, establishing predictive models to identify optimal beneficiary populations, and performing cost-effectiveness analyses will provide higher-level evidence for the clinical application of this technology.

## 5 Conclusion

Overall, this preliminary, uncontrolled case series observed improvements in swallowing function and vocal cord mobility, and reductions in medical device dependence following a combined intervention protocol in patients with brain injury. However, given the absence of a control group, the wide range of injury-to-enrollment duration (32.8–178.0 days), and the concurrent delivery of multiple active interventions, it is not possible to attribute these improvements to any specific component of the protocol or to exclude a meaningful contribution from spontaneous neurological recovery. Findings should be regarded as exploratory and hypothesis-generating only, and must be interpreted with caution regarding internal validity.

Although the study design limitations prevent establishment of causal relationships, the study results provide clinicians with new therapeutic approaches and lay the foundation for subsequent high-quality clinical trials to promote evidence-based development of this technology.

## Data Availability

The datasets presented in this article are not readily available because of ethical and privacy restrictions. Requests to access the datasets should be directed to the corresponding authors.
